# Cloud designs and deployment models: a systematic mapping study

**DOI:** 10.1186/s13104-019-4474-y

**Published:** 2019-07-19

**Authors:** Isaac Odun-Ayo, Rowland Goddy-Worlu, Victoria Samuel, Victor Geteloma

**Affiliations:** Department of Computer and Information Sciences, Covenant University, Ota, Nigeria

**Keywords:** Cloud, Federated, Aggregator, Broker, Mapping, Service deployment, Security

## Abstract

**Background:**

Cloud computing is a unique paradigm that is aggregating resources available from cloud service providers for use by customers on demand and pay per use basis. There is a Cloud federation that integrates the four primary Cloud models and the Cloud aggregator that integrates multiple computing services. A systematic mapping study provides an overview of work done in a particular field of interest and identifies gaps for further research.

**Objectives:**

The objective of this paper was to conduct a study of deployment and designs models for Cloud using a systematic mapping process. The methodology involves examining core aspect of the field of study using the research, contribution and topic facets.

**Results:**

The results obtained indicated that there were more publications on solution proposals, which constituted 41.98% of papers relating to design and deployment models on the Cloud. Out of this, 5.34% was on security, 1.5% on privacy, 6.11% on configuration, 7.63% on implementation, 11.45% on service deployment, and 9.92% of the solution proposal was on design. The results obtained will be useful for further studies by the academia and industry in this broad topic that was examined.

**Electronic supplementary material:**

The online version of this article (10.1186/s13104-019-4474-y) contains supplementary material, which is available to authorized users.

## Introduction

Cloud computing delivers services in a virtualized manner to users through the Internet. There are four primary Cloud models, which are the private, public, community and hybrid Cloud. There is also the federated Cloud, which integrates the four Cloud models into a scalable cloud platform for several purposes and supports the Cloud aggregator. Resource provisioning in federated clouds is the process of finding optimal placement schemes for virtual machines and reconfiguring them according to changes in the environment [1]. The various cloud service providers (CSPs) use massive data centers with thousands of servers, allowing data to be replicated in data centers [2]. An alternative is suggested in [3] with many micro data centers, each interconnected by medium to high bandwidth link which is suitable for use by private and public clouds. Cloud aggregation and cloud brokerage are vital in the design and deployment of Cloud models. The goal of Cloud service brokerage is to make the service more specific to a company, to integrate and aggregate services with a view to adding value to original Cloud services being offered [4].

Infrastructure-as-a-Service (IaaS) Cloud deployment models are defined as public, private, hybrid, broker and federation [5]. Public deployment is used by any Cloud consumer over the internet, while private deployment models involves one organization [5]. Broker deployment involves the management of different transactions between multiple public Cloud and any Cloud consumer over the internet.

From the foregoing, there are several publications and work that has been carried out in the area of designs and deployment models for Cloud computing. It therefore becomes essential to have an indication of what has been done by researchers [6]. This overview can be summarized using a systematic mapping process, which provides a platform for categorizing publications based on a unique scheme and structure [7].

## Main text

### Methods

#### The systematic mapping process

The systematic mapping process for this study was carried out using the guidelines found in [8, 9]. This process, which is a repeatable one, was utilized for extracting and interpreting available materials that are based on the research objective [10]. All the stages required for conducting a systematic mapping study was applied in creating the systematic map of design and deployment models for Cloud in terms of private, public, hybrid, federated and aggregated clouds.

#### Definition of research questions

A systematic map provides an insight into the quality of work that has been done in a specific knowledge domain, which is essential in identifying areas where papers have been published. In this paper, the following are the defined research questions.

- RQN1: What are the discussed research areas on design and deployment models for Cloud in terms of private, public, hybrid, federated and aggregated Clous, and what number of papers are covered in these areas?
- RQN2: What sort of papers are issued in the research area and what are their constituted evaluation and novelty?

#### Conduct of search for primary studies

Four major electronic digital libraries (ACM, IEEE Xplore, Science direct and Springer) were utilized in the search for primary studies due to the high impact factor of their conference and journal papers. For this study on design and deployment models for Cloud, the keyword utilized in the search string was taken from every aspects of the topic of this study, which ensured the inclusion of studies relevant to the research area. The search string is as follows:

(TITLE (design AND deployment)) AND TITLE-ABS-KEY (models) AND (TITLE (Cloud) AND (TITLE-ABS-KEY (private OR public OR hybrid OR federated OR aggregated)).

#### The inclusion and exclusion criteria

The inclusion and exclusion criteria were utilized in excluding irrelevant papers that did not contribute to answering the research questions as regards this study on design and deployment models for Cloud. Publications that do not relate to the research questions or abstract, papers on editorials, panel discussions, presentation slides, prefaces, summaries and tutorials were excluded during this process.

#### Keywording of abstracts

Keywords from different papers on design and deployment models on Cloud were combined to provide a satisfactory understanding of the types and contributions made on each paper. The systematic process has the following stages [8]:

- Abstract
- Keywording
- Classification scheme
  - Articles
  - Sorting articles into scheme
  - Updating scheme
- Systematic map

This process was used to determine the categories or facets utilized in this study. Of the three facets utilized in this study, the topics facet focused on the important topics relating to the field of study obtained during the classification scheme, the contribution facet provided information on the type of contribution employed in publications related to this study, and the research facet dwelled on the kind of research conducted.

#### Research facet with categories and descriptions

The classification approaches in [10] were adopted in this study and these approaches is categorized based on the following description as shown below:

- Solution proposal: These papers solve a problem by providing a unique solution. The benefits and the disadvantages of applying the solution are also highlighted.
- Validation research: The procedure used is unique but not yet implemented either in terms of a laboratory experiment or an application as proof of concept.
- Evaluation research: The procedure has been implemented and the resultant outcomes are outlined based on pros and con.
- Experience papers: These papers relies on the knowledge of the researchers. It provides an overview of how something was done.
- Opinion papers: The study represents the perspective of the researcher.
- Philosophical papers: These papers provide a new way of identifying and solving a particular problem.

The classification approaches were deemed acceptable for use in this study and the primary studies in this work were classified based on the categories and description above in determining the research facet.

#### Data extraction and mapping of studies

For this study, the process of extracting data was done on a Microsoft Excel table, which contained a combination of the frequencies of publications in each category of the classification scheme. The frequencies were combined into tables containing either the topics and contribution facets or topics and research facets. Using the generated data on the excel table, bubble plots were created and centered on projecting the frequencies of the publications, which aided in identifying the research areas that were emphasized more in this study.

The systematic map was created using a two x–y scatter plot with bubbles at the intersection of the combined categories and the size of the bubbles corresponding to the number of papers in each category. The map was created with two quadrants whereby each quadrant offers a visual map on the combined categories, thus making it easy to examine the quadrants simultaneously.

### Result

#### Contribution category

**Fig. 1:**
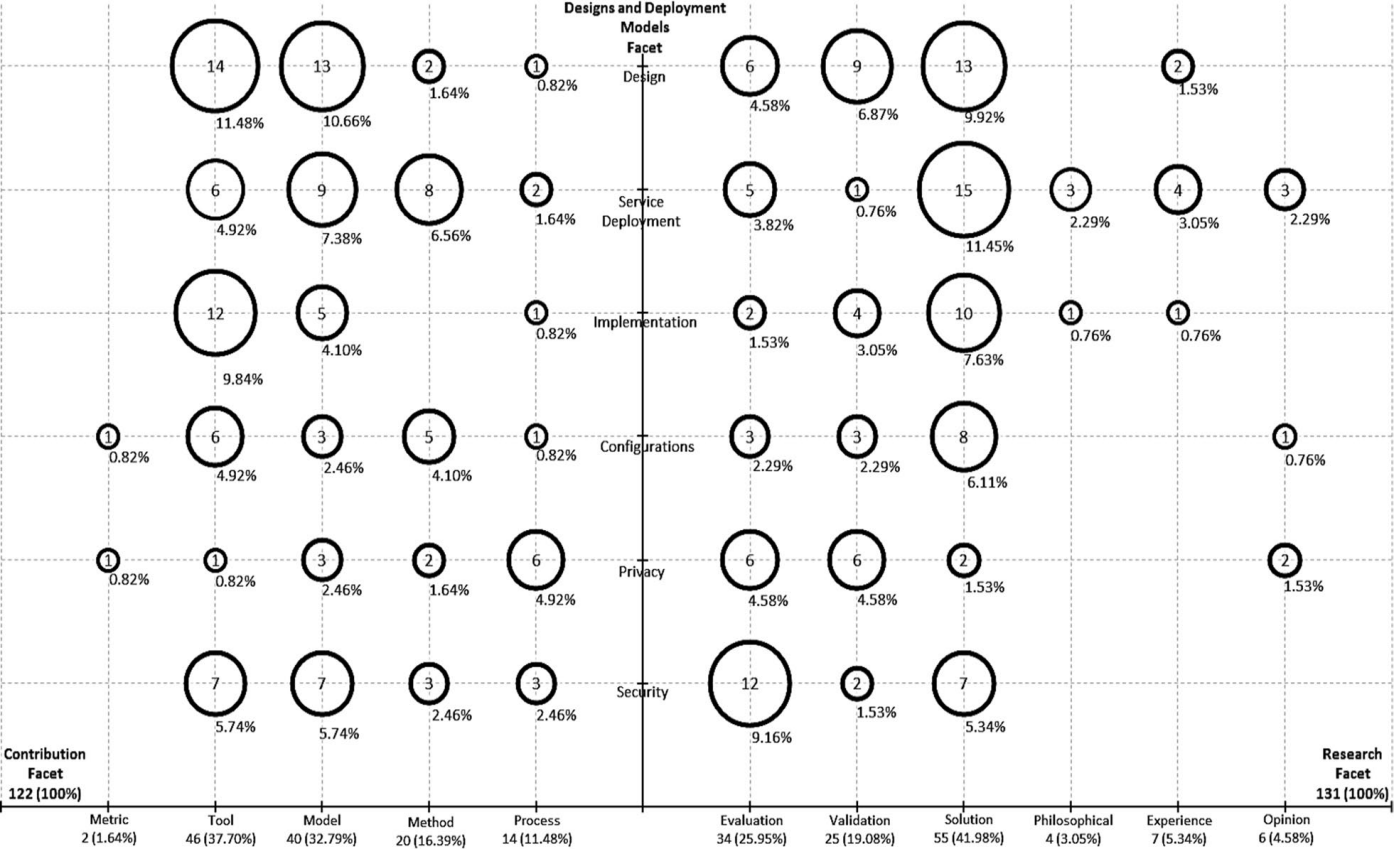
Systematic map of designs and deployment models for cloud

Figure 1 shows the systematic map of designs and deployment models for Cloud with focus on private, public, hybrid, federated and aggregated Cloud, while on Table 1 is the primary studies listed for examining the topics against the contribution facets. The result showed that out of 122 papers included in this category, 37.7% focused on tools in relation to the field of the study. Also, metrics had 1.64%, models had 32.79%, methods had 16.39% and processes had 11.48%.

**Table 1:** Topic and contribution primary studies

Topic	Contribution facet				
	Metric	Tool	Model	Method	Process
Design		11, 5, 7, 35, 37, 59, 62, 81, 90, 98, 117, 118, 122, 130	2, 3, 15, 21, 22, 23, 29, 31, 41, 42, 51, 70, 71,	54, 63	74
Service development		1, 4, 6, 56, 57, 131	13, 14, 16, 17, 44, 45, 119, 120, 121	20, 30, 34, 72, 79, 82, 94, 97	36, 61
Implementation		9, 26, 27, 33, 38, 53, 65, 83, 87, 95, 125, 128	46, 48, 52, 75, 102,		108,
Configurations	8	10, 24, 55, 64, 80, 89	8, 67, 126	68, 76, 77, 92, 103	109
Privacy	66	114	58, 60, 116	111, 123	19, 43, 100, 101, 124, 127
Security		12, 18, 69, 73, 99, 105, 113	25, 28, 47, 49, 107, 110, 112	50, 84, 96	32, 86, 93
Percentage	1.64%	37.70%	32.79%	16.39%	11.48%

#### Research type category

Shown on Table 2 is primary studies listed for examining the topics against the research facets. The result showed that out of 131 papers included in this category, 41.98% of the articles were centered on solution proposal in relation to the field of the study. Also, evaluation research was 18.32%, validation research was 26.72%, philosophical was 3.05%, experience was 5.34% and opinion papers constituted 4.58%.

**Table 2:** Topic and research primary studies

Topic	Research facet					
	Evaluation	Validation	Solution	Philosophical	Experience	Opinion
Design	5, 21, 22, 41, 42, 51	11, 15, 23, 29, 37, 59, 70, 71, 98	2, 3, 31, 35, 62, 63, 74, 81, 90, 117, 118, 122, 130		7, 54	
Service deployment	36, 72, 79, 82, 85	39	1, 4, 6, 17, 20, 30, 40, 44, 45, 56, 57, 61, 97, 104, 131	14, 16, 129,	91, 94, 120, 121,	13, 34, 119
Implementation	27, 53	33, 65, 108, 125	9, 26, 48, 52, 75, 83, 87, 95, 102, 128	46	38	
Configurations	55, 64, 76,	10, 67, 126	8, 24, 68, 77, 89, 92, 109, 103,			80
Privacy	66, 78, 106, 111, 114, 123,	43, 60, 100, 101, 124, 127	58, 116			19, 115
Security	25, 28, 32, 47, 50, 69, 73, 84, 86, 93, 107, 110	49, 112,	12, 18, 88, 96, 99, 105, 113			
Percentage	25.95%	19.08%	41.98%	3.05%	5.34%	4.58%

#### Topic and contribution facet

The following topics were extracted during the classification scheme in the area of designs and deployment for Cloud:

- Design.
- Service deployment.
- Implementation.
- Configurations.
- Privacy.
- Security.

From Fig. 1, the left quadrant shows the relationship between the topics and the contribution facet. Discussion on tool as it relates to the focus of study contributed 37.7% overall, but 5.74% were on security, 0.82% were on privacy, 4.92% were on service deployment and 11.48% were on designs. Other aspects relating to the study as regards the contribution facets is shown on Fig. 1.

#### Topic and research facet

From Fig. 1, the right quadrant combines the topic and research facets. Solution proposals constituted 41.98% of the papers in relation to design and deployment models on the Cloud. Out of this, 5.34% was on security, 1.5% on privacy, 6.11% on configuration, 7.63% on implementation, 11.45% on service deployment, and 9.92% of the solution proposal was on design. Other aspects relating to the study as regards the research facets is shown on Fig. 1.

### Discussion

#### Systematic map on policy languages and programming

The systematic map for this study comprises of two quadrants, which depicts a two x–y scatter chart with bubbles at the intersection of the topic and contribution facet, and the topic and research facet.

From the left quadrant of Fig. 1, it can be observed that there were more publications on design and service deployment in terms of tools at 11.48% and 10.86% respectively. There were more publications on service deployment in terms of methods at 6.56% and more publication on privacy in terms of processes at 4.92%. Similarly, on the right quadrant there were more publications on evaluation research in terms of security which was 9.16%. There were more publications on validation research in terms of designs at 6.87%, more articles on service deployment in terms of solution proposal at 11.45%. Also, there were more publications on service deployment in terms of philosophical, experience and opinion research with 2.29%, 3.05% and 2.29% respectively.

On the other hand, to the best of our knowledge, there were no publications based on this study on metrics as it relates to design, service deployment, implementation and security. There were also no articles on implementation in terms of methods. There were no papers on philosophical and experience research on the topics of security, privacy and configuration. Furthermore, articles on metric, model, method and tool in terms of privacy were the least at 0.82%, 0.82%, 2.46% and 1.64% respectively. It can also be identified that publications on evaluation, philosophical and experience research on the topic of implementation were the least at 1.53%, 0.76% and 0.76% respectively. In addition, the map also identified that the papers on tools, models and solutions research were emphasized more in terms of this area of study. The systematic map showed areas that lack papers coverage especially as it relates to metric, and the aspects of philosophical, experience, and opinion research. It is therefore recommended that new researchers could investigate the areas of design, service deployment and security in terms of metrics which could further provide more insights into this field. In addition, the aspects of philosophical, experience, and opinion research as it relates to security in cloud needs special focus. The papers to be written should be published in notable outlets to enable a wider reach to other researchers. In addition, new researchers could extend the electronic databases to be utilized for their studies beyond those used in this paper

## Limitations

A total of 131 papers were utilized for this study, out of an initial search comprising 1392 papers (Additional file 1).

## Additional file

**Additional file 1: Appendix S1.** Cloud designs and deployment models – Primary studies. Data contains various aspects of cloud designs and deployment models used in the analysis.

## Data Availability

The datasets used during the current study are available from the corresponding author on reasonable request.

## Abbreviations

IaaS: Infrastructure-as-a-Service
CSP: cloud service provider
ACM: Association For Computing Machinery
IEEE: Institute of Electrical and Electronics Engineers
