# Supplementary material for: Cloud designs and deployment models: a systematic mapping study
Source: BMC Res Notes. 2019 Jul 19;12:436. doi: 10.1186/s13104-019-4474-y (PMC6642572; doi:10.1186/s13104-019-4474-y)
Supplement: Supplementary file 1 — Additional file 1: Appendix S1. Cloud designs and deployment models – Primary studies. Data contains various aspects of cloud designs and deployment models used in the analysis. [file 13104_2019_4474_MOESM1_ESM.docx]

Cloud designs and deployment models – Primary studies

1. Aljahdali, H., Townend, P., Xu, J. Enhancing multi-tenancy security in the cloud IaaS model over public deployment (2013) Proceedings - 2013 IEEE 7th International Symposium on Service-Oriented System Engineering, SOSE 2013, art. no. 6525550, pp. 385-390.
2. Andreadis, G., Fourtounis, G., Bouzakis, K.-D. Collaborative design in the era of cloud computing (2015) Advances in Engineering Software, 81 (C), pp. 66-72.
3. Aversa, R., Tasquier, L. Design of an agent based monitoring framework for federated clouds (2016) Proceedings - IEEE 30th International Conference on Advanced Information Networking and Applications Workshops, WAINA 2016, art. no. 7471183, pp. 115-120.
4. Avramova, A.P., Christiansen, H.L., Iversen, V.B. Cell deployment optimization for cloud radio access networks using teletraffic theory (2015) AICT 2015 - 11th Advanced International Conference on Telecommunications, pp. 96-101.
5. Bai, T.D.P., Rabara, S.A. Design and Development of Integrated, Secured and Intelligent Architecture for Internet of Things and Cloud Computing (2015) Proceedings - 2015 International Conference on Future Internet of Things and Cloud, FiCloud 2015 and 2015 International Conference on Open and Big Data, OBD 2015, art. no. 7300911, pp. 817-822.
6. Benson, J.O., Prevost, J.J., Rad, P. Survey of automated software deployment for computational and engineering research (2016) 10th Annual International Systems Conference, SysCon 2016 - Proceedings, art. no. 7490666, .
7. Bhardwaj, A., Subrahmanyam, G.V.B., Avasthi, V., Sastry, H. Design a resilient network infrastructure security policy framework (2016) Indian Journal of Science and Technology, 9 (19), art. no. 90133, .
8. Bianchi, I., de Sousa, R.D. IT governance for public universities: Proposal for a framework using Design Science Research (2015) Espacios, 36 (21), art. no. E3, .
9. Brunner, S., Blochlinger, M., Toffetti, G., Spillner, J., Bohnert, T.M. Experimental Evaluation of the Cloud-Native Application Design (2015) Proceedings - 2015 IEEE/ACM 8th International Conference on Utility and Cloud Computing, UCC 2015, art. no. 7431462, pp. 488-493.
10. Chen, Y.-S., Lin, C.-K., Chuang, H.-M., Cheng, M.-C. Design of a hybrid model for dynamic engagement behaviour analysis in a cloud-based environment (2017) International Journal of Ad Hoc and Ubiquitous Computing, 25 (1-2), pp. 85-96.
11. Correa, J., Garcia, J. Software for optimization and time reduction in substation design, using BIM Technologies, advanced information systems and knowledge management. (2014) CIGRE Session 45 - 45th International Conference on Large High Voltage Electric Systems 2014, 2014-August, .
12. Demchenko, Y., Belloum, A., Laat, C.D., Loomis, C., Wiktorski, T., Spekschoor, E. Customisable data science educational environment: From competences management and curriculum design to virtual labs on-demand (2017) Proceedings of the International Conference on Cloud Computing Technology and Science, CloudCom, 2017-December, pp. 363-368.
13. Di Martino, B., Esposito, A., Cretella, G. From business process models to cloud deployment: A semantic approach (2016) Proceedings - IEEE 30th International Conference on Advanced Information Networking and Applications Workshops, WAINA 2016, art. no. 7471184, pp. 121-126.
14. Distefano, S., Merlino, G., Puliafito, A. Application deployment for IoT: An infrastructure approach (2013) GLOBECOM - IEEE Global Telecommunications Conference, art. no. 6831498, pp. 2798-2803.
15. Dollmann, T., Loos, P., Fellmann, M., Thomas, O., Hoheisel, A., Katranuschkov, P., Scherer, R.J. Design and usage of a process-centric collaboration methodology for virtual organizations in hybrid environments (2011) International Journal of Intelligent Information Technologies, 7 (1), pp. 45-64.
16. Dumke, L.R., Mueller, J.C. ITS deployment and improvements in smaller cities: Minnesota case studies (2004) TAC/ATC 2004 - 2004 Annual Conference and Exhibition of the Transportation Association of Canada: Transportation Innovation - Accelerating the Pace, .
17. Eid, M.I.M., Al-Jabri, I.M., Sohail, M.S. An expert system approach for the selection of cloud service delivery and deployment models (2017) Proceedings of the 10th IADIS International Conference on Information Systems 2017, IS 2017, pp. 133-144.
18. Epelde, G., Mujika, A., Leškovský, P., de Mauro, A. Public access architecture design of an FPGA-hardware-based nervous system emulation remote lab (2015) Advances in Intelligent Systems and Computing, 369, pp. 559-569.
19. Fatema, K., Healy, P.D., Emeakaroha, V.C., Morrison, J.P., Lynn, T. A data location control model for cloud service deployments (2015) Communications in Computer and Information Science, 512, pp. 117-133.
20. Fatih, G., Hidayanto, A.N., Alfina, I., Purwandari, B. Framework for selecting cloud deployment model in government institutions using BCOR, entropy and TOPSIS approach (2015) International Journal of Innovation and Learning, 18 (1), pp. 81-100.
21. Gadhavi, L., Bhavsar, M., Bhatnagar, M., Vasoya, S. Design of efficient algorithm for secured key exchange over Cloud Computing (2016) Proceedings of the 2016 6th International Conference - Cloud System and Big Data Engineering, Confluence 2016, art. no. 7508110, pp. 180-187.
22. Ge, L., Xu, X. A scheme design of cloud + end technology in demand side management (2015) American Journal of Engineering and Applied Sciences, 8 (4), pp. 736-747.
23. Gesvindr, D., Buhnova, B. Architectural tactics for the design of efficient PaaS cloud applications (2016) Proceedings - 2016 13th Working IEEE/IFIP Conference on Software Architecture, WICSA 2016, art. no. 7516825, pp. 158-167.
24. Girma, A., Abayomi, K., Garuba, M. The design, data flow architecture, and methodologies for a newly researched comprehensive hybrid model for the detection of DDoS attacks on cloud computing environment (2016) Advances in Intelligent Systems and Computing, 448, pp. 377-387.
25. Gkiokas, A., Tsardoulias, E.G., Mitkas, P.A. Hive collective intelligence for cloud robotics: A hybrid distributed robotic controller design for learning and adaptation (2015) Advances in Intelligent Systems and Computing, 351, pp. 65-78.
26. Gmach, D., Rolia, J., Cherkasova, L. Resource and virtualization costs up in the cloud: Models and design choices (2011) Proceedings of the International Conference on Dependable Systems and Networks, art. no. 5958252, pp. 395-402.
27. Gomathi, S., Venkatesan, T., Sri Vidhya, D. Design and Implementation of Fault Current Limiters in Distribution System Using Internet of Things (2018) Wireless Personal Communications, pp. 1-24. Article in Press.
28. Habbal, A., Hassan, S., Addokali, B.M., Benamar, N. Design and assessment of an experimental SDN-enabled private cloud using Openstack (2017) Journal of Telecommunication, Electronic and Computer Engineering, 9 (1-4), pp. 1-5.
29. Hamel, P., Adam, J.-P., Kubické, G., Pouliguen, P. Design of a stealth wind turbine (2012) LAPC 2012 - 2012 Loughborough Antennas and Propagation Conference, art. no. 6403021, .
30. Han, K., Cai, X., Rong, H. An Evolutionary Game Theoretic Approach for Efficient Virtual Machine Deployment in Green Cloud (2016) Proceedings - 2015 International Conference on Computer Science and Mechanical Automation, CSMA 2015, art. no. 7371551, pp. 1-4.
31. Harter, I.B.B., Hoffmann, M., Schupke, D.A., Carle, G. Scalable resilient virtual network design algorithms for cloud services (2014) Proceedings of 2014 6th International Workshop on Reliable Networks Design and Modeling, RNDM 2014, art. no. 7014941, pp. 123-130.
32. Hayashi, M., Matsumoto, N., Nishimura, K., Tanaka, H. Design of network resource federation towards future open access networking (2011) AICT 2011 - 7th Advanced International Conference on Telecommunications, pp. 130-134.
33. He, P., Qiu, J., Yi, Y., Cai, J., Dai, Z. Design of trust model based on cloud computing (2015) ISME 2015 - Proceedings of the Information Science and Management Engineering III, pp. 100-104.
34. Head, M.R., Sailer, A., Shaikh, H., Shea, D.G. Towards self-assisted troubleshooting for the deployment of private clouds (2010) Proceedings - 2010 IEEE 3rd International Conference on Cloud Computing, CLOUD 2010, art. no. 5557997, pp. 156-163.
35. Hsu, P.-F., Ray, S., Li-Hsieh, Y.-Y. Examining cloud computing adoption intention, pricing mechanism, and deployment model (2014) International Journal of Information Management, 34 (4), pp. 474-488.
36. Hu, J., Zhang, Y., Huang, C. Design of access control in E-government system based on cloud platforms (2013) Huazhong Keji Daxue Xuebao (Ziran Kexue Ban)/Journal of Huazhong University of Science and Technology (Natural Science Edition), 41 (SUPPL.2), pp. 147-151.
37. Huang, F., Li, H., Yuan, Z., Li, X. An Application Deployment Approach Based on Hybrid Cloud (2017) Proceedings - 3rd IEEE International Conference on Big Data Security on Cloud, BigDataSecurity 2017, 3rd IEEE International Conference on High Performance and Smart Computing, HPSC 2017 and 2nd IEEE International Conference on Intelligent Data and Security, IDS 2017, art. no. 7980320, pp. 74-79.
38. Huang, Q., Lou, X., Wang, W., Ni, S. Research and design for TPL-LIP based on hybrid cloud architecture (2012) International Journal of Advancements in Computing Technology, 4 (20), pp. 421-431.
39. Indriani, W., Budi, N.F.A., Azzahro, F., Hidayanto, A.N., Solikin Selection of cloud deployment model for Ministry of Foreign Affairs using Benefit, Cost, Opportunity, and Risk (BCOR) Analysis and Analytic Hierarchy Process (AHP) (2017) 2016 International Conference on Informatics and Computing, ICIC 2016, art. no. 7905761, pp. 447-452.
40. Jaatun, M.G., Zhao, G., Vasilakos, A.V., Nyre, Å.A., Alapnes, S., Tang, Y. The design of a redundant array of independent net-storages for improved confidentiality in cloud computing (2012) Journal of Cloud Computing, 1 (1), pp. 1-19.
41. Jackson, S.J., Gillespie, T., Payette, S. The policy knot: Re-integrating policy, practice and design in CSCW studies of social computing (2014) Proceedings of the ACM Conference on Computer Supported Cooperative Work, CSCW, pp. 588-602.
42. Jain, S., Peddoju, S.K. Prediction model for suitability of resource deployment using complex templates in OpenStack (2017) Proceeding - IEEE International Conference on Computing, Communication and Automation, ICCCA 2017, 2017-January, pp. 835-840.
43. Javed, A., Larijani, H., Ahmadinia, A., Emmanuel, R., Mannion, M., Gibson, D. Design and Implementation of a Cloud Enabled Random Neural Network-Based Decentralized Smart Controller with Intelligent Sensor Nodes for HVAC (2017) IEEE Internet of Things Journal, 4 (2), art. no. 7740096, pp. 393-403.
44. Jiao, H.-J., Zhang, J., Li, J.-H., Shi, J.-F., Li, J. Cloud workflow model for collaborative design based on hybrid petri net (2014) Yingyong Kexue Xuebao/Journal of Applied Sciences, 32 (6), pp. 645-651.
45. Junfeng, T., Weiping, L. Design and research of hybrid network electronic forensics model based on cloud computing (2016) International Journal of Grid and Distributed Computing, 9 (12), pp. 75-86.
46. Kaushal, U., Sharma, R. An integrated cloud computing based framework for new application and deployment model of e-governance for higher education in India (2016) E-Governance in India: Problems, Prototypes and Prospects, pp. 137-146.
47. Kaviani, N., Wohlstadter, E., Lea, R. Partitioning of web applications for hybrid cloud deployment (2014) Journal of Internet Services and Applications, 5 (1), pp. 1-17.
48. Keung, J., Kwok, F. Cloud deployment model selection assessment for SMEs: Renting or buying a cloud (2012) Proceedings - 2012 IEEE/ACM 5th International Conference on Utility and Cloud Computing, UCC 2012, art. no. 6424925, pp. 21-28.
49. Khan, K.A., Wang, Q., Luo, C., Wang, X., Grecos, C. Impact of different cloud deployments on real-time video applications for mobile video cloud users(2015) Proceedings of SPIE - The International Society for Optical Engineering, 9400, art. no. 94000P, .
50. Knittl, S., Brenner, M. Towards a configuration management system for hybrid cloud deployments (2011) Proceedings of the 12th IFIP/IEEE International Symposium on Integrated Network Management, IM 2011, art. no. 5990508, pp. 1074-1077.
51. Ko, R.K.L., Tan, A.Y.S., Ng, G.P.Y. 'Time' for Cloud? Design and implementation of a time-based cloud resource management system (2014) IEEE International Conference on Cloud Computing, CLOUD, art. no. 6973783, pp. 530-536.
52. Kuehlmann, A., Camposano, R., Colgan, J., Chilton, J., George, S., Griffith, R., Leventis, P., Singh, D. Does IC design have a future in the clouds? (2010) Proceedings - Design Automation Conference, pp. 412-414.
53. Kwon, H.-K., Seo, K.-K. Development of a hybrid life cycle cost model for estimating product design alternatives in cloud computing based collaborative design environment (2013) Advanced Materials Research, 658, pp. 614-619.
54. Lee, Y.-C., Tang, H.N., Sugumaran, V. A deployment model for cloud computing using the analytic hierarchy process and BCOR analysis (2012) 18th Americas Conference on Information Systems 2012, AMCIS 2012, 6, pp. 4247-4256.
55. Leitner, P., Cito, J., Stöckli, E. Modelling and managing deployment costs of microservice-based cloud applications (2016) Proceedings - 9th IEEE/ACM International Conference on Utility and Cloud Computing, UCC 2016, pp. 165-174.
56. Lenk, A., Dänschel, C., Klems, M., Bermbach, D., Kurze, T. Requirements for an IaaS deployment language in federated Clouds (2011) Proceedings - 2011 IEEE International Conference on Service-Oriented Computing and Applications, SOCA 2011, art. no. 6166249,
57. Li, D., Gong, Y., Shen, N., Qin, T. Design and security strategy analysis for SaaS and private cloud-based OA platform (2012) Advances in Intelligent and Soft Computing, 136, pp. 251-258.
58. Li, H., Luo, X. Design of evolutionary algorithm for the optimization of cloud storage deployment (2013) Dongnan Daxue Xuebao (Ziran Kexue Ban)/Journal of Southeast University (Natural Science Edition), 43 (SUPPL.I), pp. 202-205.
59. Li, J.Z., Woodside, M., Chinneck, J., Litiou, M. Adaptive Cloud Deployment Using Persistence Strategies and Application Awareness (2017) IEEE Transactions on Cloud Computing, 5 (2), pp. 276-289.
60. Li, N., Yi, W., Bi, Z., Kong, H., Gong, G. An optimisation method for complex product design (2013) Enterprise Information Systems, 7 (4), pp. 470-489.
61. Li, W., Svärd, P., Tordsson, J., Elmroth, E. A general approach to service deployment in cloud environments (2012) Proceedings - 2nd International Conference on Cloud and Green Computing and 2nd International Conference on Social Computing and Its Applications, CGC/SCA 2012, art. no. 6382792, pp. 17-24.
62. Lin, C., Yang, J. Cost-efficient Deployment of Fog Computing Systems at Logistics Centers in Industry 4.0 (2018) IEEE Transactions on Industrial Informatics, . Article in Press.
63. Lin, H., Xu, L., Huang, X., Wu, W., Huang, Y. A trustworthy access control model for mobile cloud computing based on reputation and mechanism design (2015) Ad Hoc Networks, 35, pp. 51-64.
64. Lin, J., Lee, M.R., Chang, T., Yang, S. A cloud deployment approach for consumer support systems (2014) Proceedings - Pacific Asia Conference on Information Systems, PACIS 2014,
65. Liqun, L., Sijin, H. A memory architecture design for high-performance cloud computing (2012) Advanced Materials Research, 532-533, pp. 677-681.
66. Lu, H.T., Kao, C.H., Wu, P.H., Yang, C.C., Chi, P.H. Design and Implementation of HPC-SA in OpenStack Cloud Platform (2016) Proceedings of International Conference on Computational Intelligence, Modelling and Simulation, 2016-September, art. no. 7579695, pp. 55-60.
67. Lu, P., Zhou, D., Chen, Q., Zhong, S. Design of the public service platform in digital campus based on "cloud + agent" mode (2012) International Journal of Digital Content Technology and its Applications, 6 (18), pp. 418-426.
68. Lu, X., Zhang, M.-Q. Design of campus cloud model based on telecom public service platform (2011) Proceedings - 2011 IEEE International Conference on Computer Science and Automation Engineering, CSAE 2011, 1, art. no. 5953203, pp. 199-202.
69. Mai, V., Khalil, I. Design and implementation of a secure cloud-based billing model for smart meters as an Internet of things using homomorphic cryptography (2017) Future Generation Computer Systems, 72, pp. 327-338.
70. Mathias, M., Velay, X., Wade, R. The challenges of assessing digital product design (2008) DS 46: Proceedings of E and PDE 2008, the 10th International Conference on Engineering and Product Design Education, pp. 6P.
71. McEvoy, G.V., Schulze, B., Garcia, E.L.M. Performance and deployment evaluation of a parallel application on a private Cloud (2011) Concurrency Computation Practice and Experience, 23 (17), pp. 2048-2062.
72. Melo, C., Dantas, J., Fé, I., Oliveira, A., Maciel, P. Synchronization server infrastructure: A relationship between system downtime and deployment cost (2017) 2017 IEEE International Conference on Systems, Man, and Cybernetics, SMC 2017, 2017-January, pp. 1250-1255.
73. Meng, Z. Research on design of post-earthquake rehabilitation scheme of digitized safe campuses (2013) World Information on Earthquake Engineering, 29 (3), pp. 50-53.
74. Nanig, T.T. Private cloud deployment model for academic environment using CloudStack (2016) Advances in Intelligent Systems and Computing, 387, pp. 155-164.
75. Parra, J. Phases, scaffolds, and technology: Cloud-based student collaboration model for online and blended course design (2016) Integration of Cloud Technologies in Digitally Networked Classrooms and Learning Communities, pp. 193-216.
76. Phillips, S., Duz, A., Pasqualetti, F., Sanfelice, R.G. Hybrid attack monitor design to detect recurrent attacks in a class of cyber-physical systems (2018) 2017 IEEE 56th Annual Conference on Decision and Control, CDC 2017, 2018-January, pp. 1368-1373.
77. Praveena, D., Rangarajan, P. Analysis of trend, service and deployment models in cloud computing with focus on hybrid cloud and its implementation (2014) Research Journal of Applied Sciences, 9 (4), pp. 181-186.
78. Prilepova, O., Hart, Q., Merz, J., Parker, N., Bandaru, V., Jenkins, B. Design of a GIS-based web application for simulating biofuel feedstock yields (2014) ISPRS International Journal of Geo-Information, 3 (3), pp. 929-941.
79. Qasem, A.A., Mahmoud, Q.H. Design and implementation of a framework for provisioning algorithms as a service (2017) International Journal of Cloud Computing, 6 (3), pp. 265-288.
80. Sandikkaya, M.T., Ovatman, T., Harmanci, A.E. Design and formal verification of a cloud compliant secure logging mechanism (2016) IET Information Security, 10 (4), pp. 203-214.
81. Saravanakumar, C., Arun, C. Location awareness of the cloud storage with trust management using common deployment model (2013) 2013 4th International Conference on Computing, Communications and Networking Technologies, ICCCNT 2013, art. no. 6726703, .
82. Saurabh, K., Ranjan, R. Cloud management simulation and design (2012) Proceedings - 2012 14th International Conference on Modelling and Simulation, UKSim 2012, art. no. 6205501, pp. 522-527.
83. Shaba, K., Hickey, C. A consequence based assessment method/software model to facilitate the safe location and design of occupied buildings on or in close proximity to process facilities (2013) 1st CCPS Asia-Pacific Conference on Process Safety 2013, APCPS 2013, pp. 65-82.
84. Shao, P., Ye, F., Wang, X., Guo, Y., Gao, G., Geng, J. Design of technical support system for retail company based on cloud (2017) MATEC Web of Conferences, 139, art. no. 00096, .
85. Siahos, Y., Papanagiotou, I., Georgopoulos, A., Tsamis, F., Nikoltsios, L. Implementing a hybrid cloud infrastructure to facilitate ICT in education: Design and evaluation (2014) Educational Technology Use and Design for Improved Learning Opportunities, pp. 1-18.
86. Singh, G., Singh, A.K. Design and implementation of virtual hadoop cluster on private cloud (2018) Communications in Computer and Information Science, 799, pp. 61-71.
87. Slawik, M., Zilci, B.I., Demchenko, Y., Baranda, J.I.A., Branchat, R., Loomis, C., Lodygensky, O., Blanchet, C. CYCLONE Unified Deployment and Management of Federated, Multi-cloud Applications (2015) Proceedings - 2015 IEEE/ACM 8th International Conference on Utility and Cloud Computing, UCC 2015, art. no. 7431456, pp. 453-457.
88. Song, B., Tian, Y., Zhou, B. Design and evaluation of remote video surveillance system on private cloud (2015) Proceedings - 2014 International Symposium on Biometrics and Security Technologies, ISBAST 2014, art. no. 7013131, pp. 256-262.
89. Srinivasan, M.K., Sarukesi, K., Revathy, P. ECloudIDS tier-1 iCloudIDM layer-I (iCloudIDM-LI) subsystem design and implementation through user-centric identity management approach for secure cloud computing environment (2013) Proceedings - IEEE International Conference on Mobile Data Management, 2, art. no. 6569091, pp. 206-211.
90. Srinivasan, M.K., Sarukesi, K., Keshava, A., Revathy, P. eCloudIDS - Design roadmap for the architecture of next-generation hybrid two-tier expert engine-based IDS for cloud computing environment (2012) Communications in Computer and Information Science, 335 CCIS, pp. 358-371.
91. Srinivasan, M.K., Sarukesi, K., Keshava, A., Revathy, P. eCloudIDS tier-1 uX-engine subsystem design and implementation using Self-Organizing Map (SOM) for secure cloud computing environment (2012) Communications in Computer and Information Science, 335 CCIS, pp. 432-443.
92. Stockton, D.B., Santamaria, F. Automating NEURON Simulation Deployment in Cloud Resources (2017) Neuroinformatics, 15 (1), pp. 51-70.
93. Strebel, J., Stage, A. An economic decision model for business software application deployment on hybrid Cloud environments (2010) MKWI 2010 - Multikonferenz Wirtschaftsinformatik 2010, pp. 47-48.
94. Su, H.M., Wang, L. Design on the network model of cloud computing based on P2P (2013) Advanced Materials Research, 756-759, pp. 1605-1609.
95. Su, L., Li, L., Zhang, L., Nie, X. Research and design of electric power private cloud data storage model (2012) Proceedings - 4th International Conference on Computational and Information Sciences, ICCIS 2012, art. no. 6300527, pp. 892-895.
96. Sun, W., Guo, C.J., Jiang, Z., Zhang, X., Duan, N., Huang, Y., Xiong, Y.D. Design aspects of Software as a Service to enable e-Business through Cloud platform (2010) Proceedings - IEEE International Conference on E-Business Engineering, ICEBE 2010, art. no. 5704357, pp. 456-461.
97. Sundararajan, P., Durairajan, S. Portable service management deployment over cloud platforms to support production workloads (2013) 2013 IEEE International Conference on Cloud Computing in Emerging Markets, CCEM 2013, art. no. 6684438, .
98. Suram, S., MacCarty, N.A., Bryden, K.M. Engineering design analysis utilizing a cloud platform (2018) Advances in Engineering Software, 115, pp. 374-385.
99. Thurk, F., Kampusch, S., Kaniusas, E. Management Framework for Biosignals in Biomedical Studies: From Study Design to Data Statistics (2016) IEEE Transactions on Instrumentation and Measurement, 65 (4), art. no. 7314922, pp. 776-782.
100. Venkateswaran, S., Sarkar, S. Architectural partitioning and deployment modeling on hybrid clouds (2018) Software - Practice and Experience, 48 (2), pp. 345-365.
101. Wang, D., Wang, Y., Sun, R., Zhang, X. Robust C-RAN precoder design for wireless fronthaul with imperfect channel state information (2017) IEEE Wireless Communications and Networking Conference, WCNC, art. no. 7925528, .
102. Wang, H., Leskinen, J., Lee, D.-S., Périaux, J. Active flow control of airfoil using mesh/meshless methods coupled to hierarchical genetic algorithms for drag reduction design (2013) Engineering Computations (Swansea, Wales), 30 (4), art. no. 17088373, pp. 562-580.
103. Wang, J., Qiao, L., Qie, Y. Process planning service model design for cloud manufacturing (2018) IEEE International Conference on Automation Science and Engineering, 2017-August, pp. 1169-1173.
104. Wang, J.C.-H., Leung, T.S.-H., Pigeon, C. Impinging injector design for a paraffin-nitrous oxide hybrid rocket engine used in sounding rockets Part I: CFD simulation of candidate designs (2016) Proceedings of the International Astronautical Congress, IAC, .
105. Wang, Q., Dou, Y., Cheng, L., Ke, Y. Shimming design and optimal selection for non-uniform gaps in wing assembly (2017) Assembly Automation, 37 (4), pp. 471-482.
106. Wang, S.-L., Chen, Y.L., Kuo, A.M.-H., Chen, H.-M., Shiu, Y.S. Design and evaluation of a cloud-based Mobile Health Information Recommendation system on wireless sensor networks (2016) Computers and Electrical Engineering, 49, pp. 221-235.
107. Wang, W., Xu, P., Yang, L.T., Li, H. A design for cloud-assisted Fair-Play Management System of online contests with provable security (2015) Future Generation Computer Systems, 52, art. no. 2688, pp. 137-146.
108. Wang, X., Wang, L., Elayoubi, S.E., Conte, A., Mukherjee, B., Cavdar, C. Centralize or distribute? A techno-economic study to design a low-cost cloud radio access network (2017) IEEE International Conference on Communications, art. no. 7996771, .
109. Wang, Z.-Y., Li, Q., Cao, Z.-C., Li, W.-H., Li, J., Du, R.-Y. A model-based deployment framework of integrated public cloud service (2012) Proceedings - 2012 International Conference on Computer Science and Service System, CSSS 2012, art. no. 6394424, pp. 723-728.
110. Watanabe, H., Ishihara, H., Hayashi, K., Kawazoe, F., Kikuchi, N., Eguchi, N., Matsunaga, T., Yokota, T. Detailed design of the GOSAT DHF at NIES and data acquisition/processing/ distribution strategy (2008) Proceedings of SPIE - The International Society for Optical Engineering, 7106, art. no. 71060N, .
111. Weiquan, X., Houkui, W. The design research of data security model based on public cloud (2013) Proceedings - 9th International Conference on Computational Intelligence and Security, CIS 2013, art. no. 6746501, pp. 607-609.
112. Wen, Z., Cala, J., Watson, P., Romanovsky, A. Cost Effective, Reliable and Secure Workflow Deployment over Federated Clouds (2017) IEEE Transactions on Services Computing, 10 (6), art. no. 7435330, pp. 929-941.
113. Wen, Z., Cala, J., Watson, P., Romanovsky, A. Cost Effective, Reliable, and Secure Workflow Deployment over Federated Clouds (2015) Proceedings - 2015 IEEE 8th International Conference on Cloud Computing, CLOUD 2015, art. no. 7214096, pp. 604-612.
114. Wu, D., Liu, X., Hebert, S., Gentzsch, W., Terpenny, J. Democratizing digital design and manufacturing using high performance cloud computing: Performance evaluation and benchmarking (2017) Journal of Manufacturing Systems, 43, pp. 316-326.
115. Wu, J., Li, H., Che, P. Service co-production and the value co-creation: The case for a private cloud service deployment (2015) 2015 12th International Conference on Service Systems and Service Management, ICSSSM 2015, art. no. 7170258, .
116. Xiao, W., Ji, C., Li, J. Design and implementation of massive data retrieving based on cloud computing platform (2013) Applied Mechanics and Materials, 303-306, pp. 2235-2240.
117. Xiong, Y.-H., Wan, S.-Y., He, Y., Su, D. Design and implementation of a prototype cloud video surveillance system (2014) Journal of Advanced Computational Intelligence and Intelligent Informatics, 18 (1), pp. 40-47.
118. Xue-Ni, H. University network-design based on cloud computing (2013) Proceedings - 5th International Conference on Intelligent Networking and Collaborative Systems, INCoS 2013, art. no. 6630426, pp. 300-303.
119. Yang, S.-J., Lai, P.-C., Lin, J. Design role-based multi-tenancy access control scheme for cloud services (2013) Proceedings - 2013 International Symposium on Biometrics and Security Technologies, ISBAST 2013, art. no. 6597702, pp. 273-279.
120. Yim, J. Design of a time colored Petri net model of the cloud-based mobile TV system (2015) International Journal of Software Engineering and its Applications, 9 (8), pp. 241-252.
121. Yoon, G., Kim, K., Lee, K. Design and implementation of geo-spatial image processing system using OGC WPS 2.0 and Web framework on Openstack cloud (2016) 4th International Workshop on Earth Observation and Remote Sensing Applications, EORSA 2016 - Proceedings, art. no. 7552781, pp. 132-135.
122. Yuan, Q., Chuecos, M. The design and development of a cloud-based digital gallery solution (2017) 30th International Conference on Computer Applications in Industry and Engineering, CAINE 2017, pp. 23-28.
123. Yuen, K.K.F. A hybrid fuzzy quality function deployment framework using cognitive network process and aggregative grading clustering: An application to cloud software product development (2014) Neurocomputing, 142, pp. 95-106.
124. Zafar, F., Khan, A., Malik, S.U.R., Ahmed, M., Anjum, A., Khan, M.I., Javed, N., Alam, M., Jamil, F. A survey of cloud computing data integrity schemes: Design challenges, taxonomy and future trends (2017) Computers and Security, 65, pp. 29-49.
125. Zeng, W., Yu, L., Zhao, L., Zhong, X., Wang, K. Framework design of cloud computing based environmental information disclosure platform (2013) Applied Mechanics and Materials, 263-
126. Zhang, C., Zhang, X., Wang, D., Wang, Y. Design and implementation of cloud service system for cold chain logistics of fresh agricultural products based on component integration (2016) Nongye Gongcheng Xuebao/Transactions of the Chinese Society of Agricultural Engineering, 32 (12), pp. 273-279.
127. Zhang, T., De Grande, R.E., Boukerche, A. Design and analysis of stochastic traffic flow models for vehicular clouds (2016) Ad Hoc Networks, 52, pp. 39-49.
128. Zhang, Y., Shen, Z., Wang, K., Kobayashi, F., Lin, X. Cloud-based virtual reality integrated automatic presentation script for understanding urban design concepts in the consensus process: A case study of one foundation's disaster prevention park in China (2017) International Review for Spatial Planning and Sustainable Development, 5 (1), pp. 29-44.
129. Zhou, L., Wang, X., Deng, L., Jing, Y., Li, W. Multi-path logistics cloud service composition design with trigger timetable (2015) Jisuanji Jicheng Zhizao Xitong/Computer Integrated Manufacturing Systems, CIMS, 21 (6), pp. 1617-1625.
130. Zou, C., Deng, H., Qiu, Q. Design and implementation of hybrid cloud computing architecture based on cloud bus (2013) Proceedings - IEEE 9th International Conference on Mobile Ad-Hoc and Sensor Networks, MSN 2013, art. no. 6726345, pp. 289-293.
131. Zou, T., Le Bras, R., Salles, M.V., Demers, A., Gehrke, J. ClouDiA: a deployment advisor for public clouds (2015) VLDB Journal, 24 (5), pp. 633-653.
